# Revisiting the “The Breakfast Club”: Testing Different Theoretical Models of Belongingness and Acceptance (and Social Self-Representation)

**DOI:** 10.3389/fpsyg.2020.604090

**Published:** 2021-01-18

**Authors:** Saga Pardede, Nicolay Gausel, Magnhild Mjåvatn Høie

**Affiliations:** ^1^Department of Psychosocial Health, University of Agder, Grimstad, Norway; ^2^Faculty of Health and Welfare, Østfold University College, Halden, Norway

**Keywords:** belongingness, acceptance, social, self, representation, needs, EFA, CFA

## Abstract

The current work tests different theoretical models of belongingness and acceptance as fundamental needs for human motivation. In the current study, 372 participants were presented with 52 different items measuring five different theoretical models of belongingness (with a total of 32 items) and three different theoretical models of acceptance (with a total of 20 items). In a first step, Confirmatory Factor Analysis (CFA) failed to provide support for these eight theoretical models. In a second step, we therefore applied Exploratory Factor Analysis yielding three factors, which we interpreted as communicating: (1) Belongingness, (2) Emotion-Acceptance, and (3) Social Self-Representation. In a third step, these three factors were corroborated by a CFA. We discuss how these two factors of “belongingness,” “emotion-acceptance” respond to the literature on the need to belong and be accepted, and we reflect on how ‘social self-representation’ seems to be an alternative motivation for how we present ourselves to our social relations to fulfill our needs.

## Introduction

“You see us as you want to see us – in the simplest terms, in the most convenient definitions. But what we found out is that each one of us is a brain … an athlete … a basket case … a princess and a criminal…”

– “The Breakfast Club” by John Hughes

This infamous 80s movie tells a story about five strangers forced to be in detention and have centered the belief of who they are around how they belong, are accepted, and how their self is socially presented. Based on social interactions with one another during detention, they were able to see themselves, not for how they are socially presented, but for who they truly are: as fellow human beings with a burning need for belongingness and acceptance fulfilled through their friendships. It is this realization of social connectedness motivating Brian’s declaration of the group as the “The Breakfast Club.”

As illustrated by the movie, the need to belong (e.g., Baumeister and Leary, 1995) and the need for acceptance (e.g., Rogers and Koch, 1959; Rogers, 1961) make up two essential parts of what it means to be oneself in response to our social relations (Cohen and Syme, 1985; Hagerty and Patusky, 1995; Leary et al., 2006; Leary and Cox, 2008). Despite the longstanding influence that the concepts of belongingness and acceptance have had on psychological thinking (e.g., Murray, 1938; Maslow, 1943, 1968; Rogers, 1951; McClelland, 1987; Deci and Ryan, 1991; Goodenow, 1993; Ryan, 1993; Vallerand, 1997) one should have expected an agreement on what “belongingness” and “acceptance” mean. Yet, there is no shared agreement on what is meant with “belongingness” (see Maslow, 1962; Thoits, 1982; Hagerty and Patusky, 1995) and “acceptance” (see Rogers, 1961; Greenberg, 1994; Cialdini and Goldstein, 2004) as each of the two constructs are defined differently depending on who you ask. By such, both “belongingness” and “acceptance” are presented as having different variations wowed within them. If this is true, one should expect that it should be possible to (1) differentiate among all the different variations of “belongingness” and “acceptance.” Alternatively, if they can’t be differentiated, we believe it should be possible to (2) demonstrate their *shared* similarities; that all definitions of “acceptance” and “belongingness” are universal and undifferentiated regardless of theoretical standpoint. However, if it should not be possible to differentiate them or find them to be so similar that they can form two distinct, but related constructs, we need to explore and re-think what “belonging” and “acceptance” means for theoreticians and researchers. With these ideas in mind, we followed Gausel et al.’s (2012) and Gausel et al.’s (2016) approach to “scale validation” using Confirmatory Factor Analysis (CFA).

## Two Psychological Needs for Human Motivation: Belongingness and Acceptance

The evaluation of the need to belong and the need for acceptance is largely judged in relation with one’s emotional bond with another (Baumeister and Leary, 1995; Shah, 2003). The degree of subjectively appraised need-fulfilment has a great impact on one’s psychological well-being, motivation, and functioning (e.g., Bowlby, 1969, 1973; Baumeister and Leary, 1995; Murphy et al., 2007; Cheryan et al., 2009). If the needs are fulfilled, they positively influence how people appraise themselves, how they feel about themselves and how they behave, (Gausel and Leach, 2011) and thus, contribute to a subjective sense of social connectedness with others (e.g., Rogers, 1951; McClelland, 1987; Baumeister and Leary, 1995; Vallerand, 1997) and better well-being (e.g., Cohen and Janicki-Deverts, 2009). Yet, if these needs are appraised as deprived or unfulfilled, a feeling of rejection is felt (Baumeister and Leary, 1995; Gausel and Leach, 2011) leading to a sense of worthlessness (Tangney and Dearing, 2002) and depression (Baumeister and Leary, 1995; Hagerty and Patusky, 1995). However, as these needs are fulfilled through our social bonds, the way we present ourselves socially to others (Ellemers et al., 2008) is an important aspect of how we balance our needs to belong and to be accepted up against the desired view of one’s social-image in the eyes of others (Gausel and Leach, 2011; Gausel, 2013).

As the two needs seem to incorporate various aspects of the way the self is functioning and develops, many different theoretical models of what “belonging,” and “acceptance” mean have evolved consequently. In our reading, we were able to detect what seemed to encompass at least five theoretical models of “belongingness” and three theoretical models of “acceptance.” For “belongingness” we identified a sense of “identity-proximity” (e.g., Kohut, 1971, 1977; Kohut et al., 1984), a sense of “emotion-sharing” (e.g., Lee and Robbins, 1995), a sense of “supportive-proximity” (e.g., Hill, 1987; Lazarus, 1991; Kelly and Barsade, 2001; Pickett et al., 2004), a sense of “similarities of *Self* and *Others*” (e.g., Tajfel and Turner, 1979; Turner, 1999), and a sense of “environmental-satisfaction” (e.g., Bronfenbrenner’s, 1979). For “acceptance,” we identified a sense of “usefulness” (e.g., Rogers and Koch, 1959), a sense of “satisfactory” (e.g., Hayes, 1994), and a sense of “attitude to change” (e.g., Vygotsky, 1978; Bandura, 1986; Linehan, 1993; Heard and Linehan, 1994). In the following, we will lay out these theoretical models.

## Five Different Theoretical Models of Belongingness

### Identity-Proximity: Understanding One’s Identity Through Proximity With the “Other”

According to Kohut (1971, 1977) and Kohut et al. (1984), there cannot be a cohesive self without someone else in proximity to mirror oneself in. That is, the “other” in the relationship provides a platform where one’s value can be mirrored, one can reciprocally be liked and by such experience a sense of connectedness and alikeness with the “other.” In this way, the experience of responses from the “other” is what reinstates and encapsulates the experiences of self and give rise to the emergence of self-identification in proximity with “the other.” Similarly, Baumeister and Leary (1995) argued that the subjective experience of oneself is experienced through one’s social relationship, where these relationships maintain a certain measure of belongingness depending on its proximal distance from oneself. Any shortcomings or failures can by such threaten one’s relationships, which in turn will threaten the need to belong, ultimately leading to severe feelings of isolation and alienation (Gausel and Leach, 2011), anxiety and depression (Baumeister and Leary, 1995; MacDonald and Leary, 2005). What we term “identity-proximity” is, therefore the understanding of one’s identity through the need to belong fulfilled via affiliation and relationship with the proximal “other.”

### Emotion-Sharing – Reciprocal Connectedness

It is theorized that the need to belong involves the psychological experience of social connectedness obtained through emotion sharing. According to Lee and Robbins (1995), a sense of belongingness evolves from infancy to maturity through companionship, affiliation, and connectedness. Despite not being able to empirically confirm this three-parted argumentation they convincingly argued that one’s need to belong is growingly associated with a sense of worth obtained through, not only caregivers, but also through affiliations with others, and later, relationships outside their comfort circle. The joint theme for their argumentation is that the need to belong in these stages are marked by the ability to reciprocally share emotions and thus experience mature connectedness. By such, Lee and Robbins (1995) argued that the need to belong is fulfilled through emotion-sharing and reciprocal connectedness.

### Supportive-Proximity – Emotional Support From Others

Lazarus (1991) proposed that people appraise stressful situations based on whether they think they have the resources to cope with the stressors or not. One of the key coping strategies in relation to social stressors is to check whether they have social resources needed to cope. Therefore, in the face of a stressor, people typically reach out to their social relations to receive emotional support. By such, appraising stressful situations should elicit one’s sense of belonging (or the need to reach out for emotional support) beyond significant others in order to re-appraise the stressor based on the emotional support one receives from whomever close enough to be offering support. Hill (1987) therefore theorized that one’s social affiliation motive can be structured on the aim for continuous emotional support, as one’s need for belongingness is sensitize based on the subjective experiences and cues (Kelly and Barsade, 2001; Pickett et al., 2004). The need to belong can therefore be understood as a need oriented toward emotional support from proximal others.

### Similarity of Self and Others – Social Identity

According to social-identity theory, understanding who one is, is affected by how we identify with similar others (Tajfel and Turner, 1979). According to Turner (1999), self-categorization becomes fully operational as a social identity only once an individual has identified with her/his social category. By such, one’s worth is influenced by the number of possible social groups in which one belongs. If belongingness has been obtained through allocation with a group of similar others, one will prioritize one’s group simply because oneself is a belonging member of that group. Thus, the need to belong is created through a cognitive process where one’s self-worth is dependent on a similarity of *Self* and *Others*’ as represented by a group-membership (Tajfel, 1978; Turner et al., 1987).

### Environmental-Satisfaction – Interactions and Experiences

A sense of belonging is attached to the influences of the environment one is interacting with. Bronfenbrenner’s (1979) ecological framework posits that human experiences and development tie themselves to the interactions of individuals and the events of their environment as satisfactory or unsatisfactory. Thus, belongingness based on “environmental-satisfaction” is interconnected to how one centers or attaches oneself with the overall satisfaction of an experience within their environment. This consequently motivates one’s need for participation and the influence of self-perception of belongingness, as this feeling of emotional connection within a setting can accordingly result in the feeling of rejection if unsatisfactory, or belonging if satisfactory (e.g., Lynch, 1976; Relph, 1976; Canter, 1977).

## Three Different Theoretical Models of Acceptance

### Usefulness

Rogers and Koch (1959) posits that there is an inherent need for acceptance to feel useful for others, that is, that there is a use for me in the world. Rogers connects this usefulness to the need for acceptance as a way to self-actualize, or to become a “fully functioning person” (p. 208). This actualizing tendency, where acceptance feeds individuals toward an evaluative process of appraised usefulness and worthiness, is a prerequisite to personal growth in Rogers’ eyes. The driving force behind this type of acceptance is the emphasis on how “useful” an individual feels in response to one’s self-actualizing tendency. As the need for acceptance is inherent to self-actualization, the experience of acceptance from others as ‘useful’, demonstrates a fulfilment of the need for acceptance. By ideal, this perception of oneself as “useful” is then consistent with the self-representation of the ideal-self, and accordingly creates a state of congruency toward a fully functioning, and accepted person (Rogers and Koch, 1959).

### Satisfactory

Hayes (1994) illustrates acceptance to be “experiencing events fully and without defense” (p. 30). By such, he suggests that regardless of the events being positive or negative the experience itself embraced without defense should be satisfying the need for acceptance. Naturally, in contrast to an unwanted experience and feeling toward the self in a social interaction, people are typically inclined to be more satisfied and more content based on a positive social interactions and relationships (e.g., Deci and Ryan, 2002; Nguyen et al., 2016). Nevertheless, the need for acceptance, and its fulfilment, according to Hayes (1994) should create a feeling of self-satisfaction when one relates to events in one’s life without a defensive stance.

### Attitude to Change

One’s attitude to change in relation to feedback from our social relations can form a basis for the fulfilment of the need to be accepted. For instance, when there is a lack of acceptance communicated, it will effectively influence one’s “attitude to change” to the point that if change is not initiated acceptance will be withdrawn and the need will go unfulfilled (Gausel, 2013). Indeed, it is only natural that the experience of not receiving acceptance from others will support one’s “attitude to change.” That said, a strong alternative to lack of acceptance as a motivator for change is unconditional acceptance (Rogers and Koch, 1959). This will also spur one’s attitude to change, especially in therapeutic engagement (e.g., Rogers and Koch, 1959; Linehan, 1994). Taken together, the need for acceptance and the attitude to change are focusing on interactions in everyday life, highlighting how the casual attribution of acceptance affects one’s ‘attitude to change’ to satisfy the need for acceptance (Vygotsky, 1978; Mccormick and Martinko, 2004; Betz, 2007).

## The Current Study

The goal of the current study was to test whether it was possible to differentiate among the five variations of belongingness and three variations of acceptance as we have suggested. However, if this is not feasible, we believe it should still be possible to tap into their shared similarities so that the eight variations reflect two larger “umbrella constructs” where one would be reflecting a global “belongingness” factor (incorporating the five variations of belonging), and the other a global “acceptance” factor (incorporating the three variations of acceptance). To investigate and test these two hypotheses, we followed Gausel et al. (2012, 2016) approach to “scale validation” performing various steps using CFA.

## Method

### Participants and Procedure

Three hundred eighty-seven English speaking community participants from 51 different nationalities (where English was the *de facto* and the *de jure* language for 15 of them) across the world were originally invited to a voluntary online survey titled “Social Relations” using Google forms. Out of these participants, 15 cases were deleted due to missing data of gender and nationality, making the final sample a total of 372 (229 women and 143 men, *M*_*age*_ = 38.39, SD = 12.27, age-range = 18–79).

Participants were first presented with information regarding the purpose of the study and the nature of their involvement as voluntary. Following this, they were reassured participation was completely anonymous, and that no information could be traced back to them. Participants were informed that the study contained statements about their thoughts toward being social and that there were no right or wrong answers, and that completion of the study was calculated to be around 10 min. Following this, participants were asked to fill in their demographics as reported above (i.e., gender, age, nationality) and then went on to respond to the 52 items developed to be representative of belongingness and acceptance.

### Measures

#### Five Variations of “belongingness”

Drawing on the framework as described in the introduction, 32 items were developed for this study to reflect the five variations of ‘belongingness’. (1) Identity-Proximity (five items α = 90, *M* = 5.36, SD = 1.17) “I feel that people listen to me,” “I feel like I can be myself,” “I feel that I am valued by others,” “I feel that I was recognised by others,” and “I feel respected by others.” (2) Emotion-Sharing (seven items α = 80, *M* = 5.24, SD = 0.75) “I have a need to belong,” “I feel the need to belong with others,” “I feel other people affect my behavior,” “I feel I am involved with other people,” “I feel close to other people,” “People need to feel that they belong,” and “I feel I can talk to people.” (3) Supportive-Proximity (six items α = 83, *M* = 5.99, SD = 0.19) “I feel it is important that people can come and share with me,” “It is important that people can come to me for help,” “I feel it is important that I can share with others,” “I feel is important that I can turn to people for help,” “I think it is important that people can trust me,” and “I think it is important that I can trust in people,” (4) Similarity of *Self* and *Others* (four items α = 90, *M* = 5.16, SD = 1.29) “I feel I am part of other,” “I feel I am recognised by others,” “I feel that I am important to others,” and “I feel I am acknowledged by others.” (5) Environmental-Satisfaction (ten items α = 79, *M* = 5.28, SD = 0.53) “I feel safe when I am with others,” “I find opportunities within the situation I am in,” “I feel comfortable within the place I am in,” “It is important for me to feel part of others,” “It is important that I feel part of the situation,” “I feel it is important that I feel belongingness where I am,” “It is important that in the situation I can depend on people,” “It is important that I am not being compared by others,” “I feel that I can work together with people,” and “It is important that I can share my ideas openly.”

#### Three Variations of “Acceptance”

Similarly, 20 items were developed in reflection to the three variations of “acceptance.” (1) Usefulness (five items α = 85, *M* = 4.99, SD = 0.99) “I feel that I am in control of my life,” “I feel emotionally connected to others,” “I feel connected to others,” “I get what I want emotionally from others,” and “I feel that people know who I am.” (2) Satisfactory (five items α = 65, *M* = 4.86, SD = 0.97) “I feel that I need to convince people about myself,” “I feel that I get positive feedback from others,” “I feel that I can be myself around people,” “I feel that I can show my emotions to others,” and “I feel I am allowed to express my emotions around others.” (3) Attitude (ten items α = 56, *M* = 4.66, SD = 0.56) “I feel that people listen to me,” “I feel I am safe,” “I feel I am useless,” “It is important that I often get good feedback,” “I would like for people to respect me more,” “I feel better when someone compliments me,” “I would like to feel good in response to others,” “It is important for people to see me the way I see myself,” “I feel that others are as much as worth as I am,” and “I actively look to fit in with others.” All 52 items were anchored with a seven-point intensity scale (1 = *not at all*, 7 = *very much*).

## Results

### Testing the Hypothesized 5 + 3 Structure: Confirmatory Factor Analysis

With the use of *AMOS 26* from *IBM*, we examined the hypothesized eight-factor structure (5 belongingness factors + 3 acceptance factors) with a CFA. Missing data within the questionnaire was handled using “Estimate means and intercepts” allowing for maximum-likelihood (ML) estimation (as little as 49 items of total of 19344 responses was left unanswered equaling 0.25%). No sample simulation was used. A variety of criteria and fit indices were considered to assess the model fit: Comparative Fit Index (CFI) > 0.90 adequate fit (see Bentler, 1990; Hu and Bentler, 1999; Schreiber et al., 2006), Incremental Fit Index (IFI) > 0.90 acceptable fit (Bentler and Bonett, 1980). In terms of Root Mean Square Error of Approximation (RMSEA) cut-off values.05 and.08 are acceptable and values between 0.08 and 0.1 are marginal (Browne and Cudeck, 1993; Fabrigar et al., 1999), and a *p*-value of <0.05. for χ*^2^* (Hu and Bentler, 1999; Mehmetoglu and Jakobsen, 2016). In line with the scale validation recommendations by Gausel et al. (2012, 2016), we allowed each of the eight factors to correlate, but no correlations were allowed between the error terms. To our disappointment, this model fit the data poorly as yielded by a significant chi-square, χ*^2^* (1246) = 4827.1, *p* < 0.000, (χ^2^/df = 3.87), a very low Comparative Fit Index: *CFI* = 0.698, a very low Incremental Fit Index: *IFI* = 0.702, but a relatively good Root Mean Square Error of Approximation, *RMSEA* = 0.088, with a Akaike information criterion index (AIC) of 5195.09. Although the isolated *RMSEA* value was decent, it did not conform with the other (very poor) fit indices and could therefore not be used as an overall indication of good fit (Kline, 1998; Hu and Bentler, 1999). Moreover, factor loadings ranged from λ = −0.83, *p* < 0.001–0.93, *p* < 0.001. indicating that the factors were poorly defined by their respective items (Gausel et al., 2012, 2016), with very high correlations between the eight different factors (ranging from *r* = −0.87, *p* = 0.012, to *r* = 0.97, *p* < 0.001) Due to the poor fit of the data, we must admit that we failed to receive support for our first hypothesis.

Our second hypothesis was based on the thinking that it should be possible to tap into the shared similarities of all the belongingness items so that they reflected a global “belongingness” factor (incorporating the five variations of belong), and the shared similarities of all the acceptance items so they reflected a global “acceptance” factor (incorporating the three variations of acceptance). As with the first model, we allowed the two factors to correlate, but no correlations were allowed between the error terms. However, as with the first model, this model fit the data poorly, χ*^2^* (1273) = 6214.08, *p* < 0.001, = (χ*^2^*/df = 4.88), *CFI* = 0.584, *IFI* = 0.587, *RMSEA* = 0.102, *AIC* = 6532.08. Moreover, the difference in Δ *AIC* = 1336.99 for this model as compared to the first model demonstrated that this latter model fit the data much worse than the original model. This conclusion was supported by the Δχ*^2^* (27) = 1386.98, *p* < 0.001, as well. By such, we must admit that we failed to receive support for our second hypothesis.

With the two failed models, we now decided to follow a third recommendation by Gausel et al. (2012, 2016) where all items collapse onto one factor. Hence, all 52 items were collapsed into a big, global “social need” factor. However, this solution did not fit the data either, χ^2^ (1274) = 6321.4, *p* < 0.001, (χ^2^/df = 4.96), *CFI* = 0.575, *IFI* = 0.578, *RMSEA* = 0.103, *AIC* = 6635.39. In fact, this model fit the data even worse than both, the first (Δ *AIC* = 1440.30, Δχ*^2^* (28) = 1494,30, *p* < 0.001) and the second model (Δ *AIC* = 103.31, Δχ*^2^* (1) = 107.32, *p* < 0.001).

### Exploring the Data: Exploratory Factor Analysis

Left with the realization that both hypotheses were not confirmed, but rather falsified, we saw it necessary to explore what “belonging” and “acceptance” meant to our community-participants and what they were trying to communicate to us. In a second step, we, therefore deployed an *Exploratory Factor Analysis* (*EFA*) with *SPSS 26* from *IBM*, in order to non-directionally investigate the relationships among the variables (Bryman and Cramer, 2005) and by such uncover the empirically distinct factorial structure of the participants’ responses to the items (Lord et al., 1968; Zhang and Stout, 1999). Thus, all 52-items were subjected to an *EFA* with a direct oblimin rotation. ML estimation was applied due to its advantage of providing standard errors for factor loadings, and a χ*^2^* fit-indicia of the overall goodness-of-fit was applied to evaluate the fit of the extracted factors (Fabrigar et al., 1999; Hayashi et al., 2007). As a first step, we examined the data in response to the default setting of Kaiser’s criterion eigenvalue of 1. Results yielded a significant [χ*^2^* (810) = 1489.12, *p* < 0.000] 10-factor solution after 11 iterations with several cross-loadings around 0.40 and 0.50 indicating too high of a correlation between the factors. By such, the 10 factors did not communicate any meaningful pattern of information to us thus, so we disregarded this initial factor solution.

In a second step, we decided to examine more closely the associated scree plot (Preacher and MacCallum, 2003). This visual information suggested that three factors should be retained. We, therefore, removed the most notorious cross-loadings and ran the *EFA* again, this time instructing SPSS to extract three factors. This move provided 11 items under three factors after 22 iterations where the sum of squared loadings ranged from 0.372 to 0.937 (see Table 1 for all loadings). Even though the factorial solution was significant, χ*^2^* (25) = 75.58, *p* < 0.000, all three factors meet the Kaiser’s criterion of eigenvalues greater than 1, and the three-parted factorial solution accounted for in total of 65.58% of the variance. Factor 1 had an eigenvalue of 3.60 and accounted for 32.77% of the variance. Factor 2 had an eigenvalue of 2.37 and accounted for a variance of 21.53%. Factor 3 had an eigenvalue of 1.24 and accounted for 11.28% of the variance.

**TABLE 1:** EFA three-factors.

Item	Factor		
	1	2	3
Q7 I fed the need to belong with	0.937	−0.244	–0.037
other			
Q6 I have a need to belong	0.799	−0.248	–0.004
Q26 It is important for me to	0.626	−026	0.127
feel part of others			
Q42 I feel I am allowed to	**0.414**	**0.816**	0.097
express my emotion around			
others			
Q41 I feel that I can show my	0.414	**0.746**	0.160
emotion to others			
Q36 I get what I want	**0.355**	**0.595**	–0.094
emotionally from others			
Q40 I feel that I can be myself	0.407	**0.595**	–0.014
around people			
Q47 I would like for people to	–0.024	−0.393	**0.589**
respect me more			
Q45 I feel that I am useless	–0.064	−0.368	**0.504**
Q38 I feel that I need to	0.084	−0.351	**0.422**
convince people about myself			
Q30 it is important that I am not	0.050	−0.101	**0.372**
being compared with others			

*Extraction method: maximum likehood, Rotation method: Oblimin with Kaiser normalization, loading larger than 0.30 are in bold.*

According to Karagoz and Kosterelioglu (2008), labeling factors discovered from factor analysis is rightly challenging, and the use of strongest factor loading can therefore guide us toward a conceptual understanding, allowing us to create an encapsulating, meaningful label for each factor. Factor 1, with its three items, was oriented toward a need to belong and being a part off others, thus, we thought this factor was best labeled as “Belongingness” (α = 0.83): “I feel the need to belong with others,” “I have a need to belong,” and “It is important for me to feel part of others.” Factor 2, communicated a sense of acceptance through being allowed to express and show ones emotions around others, thus we labeled this factor, with its four items, as “Emotion-Acceptance” (α = 0.87): “I feel that I can be myself around people,” “I feel that I can show my emotions to others,” “I feel I am allowed to express my emotions around others,” and “I get what I want emotionally from others.” Factor 3, listed four items reflecting a value-based need to represent oneself in response to one’s social environment, hence, we labeled this factor “Social Self-Representation” (α = 0.64): “It is important that I am not being compared with others,” “I feel that I need to convince people about myself,” “I feel that I am useless,” and “I would like for people to respect me more.”

### Confirmatory Factor Analysis Again: Testing the Newfound 3-Factorial Structure

As the results from the *EFA* suggested three factors, we followed the recommendations of van Prooijen and van der Kloot (2001) to test whether it was possible to replicate, and thus cross-validate, the newfound three-factorial structure with a *CFA*. If the factorial structure suggested by the exploratory approach was a sensible one, one should expect the factorial structure “to hold” within the same dataset when trying to confirm the factorial structure communicated by the participants in the *EFA* (e.g., van Prooijen and van der Kloot, 2001; Schmitt, 2011; Toyama and Yamada, 2012). Again, we followed the recommendations for scale-validation as suggested by Gausel et al. (2012, 2016).

**FIGURE 1:**
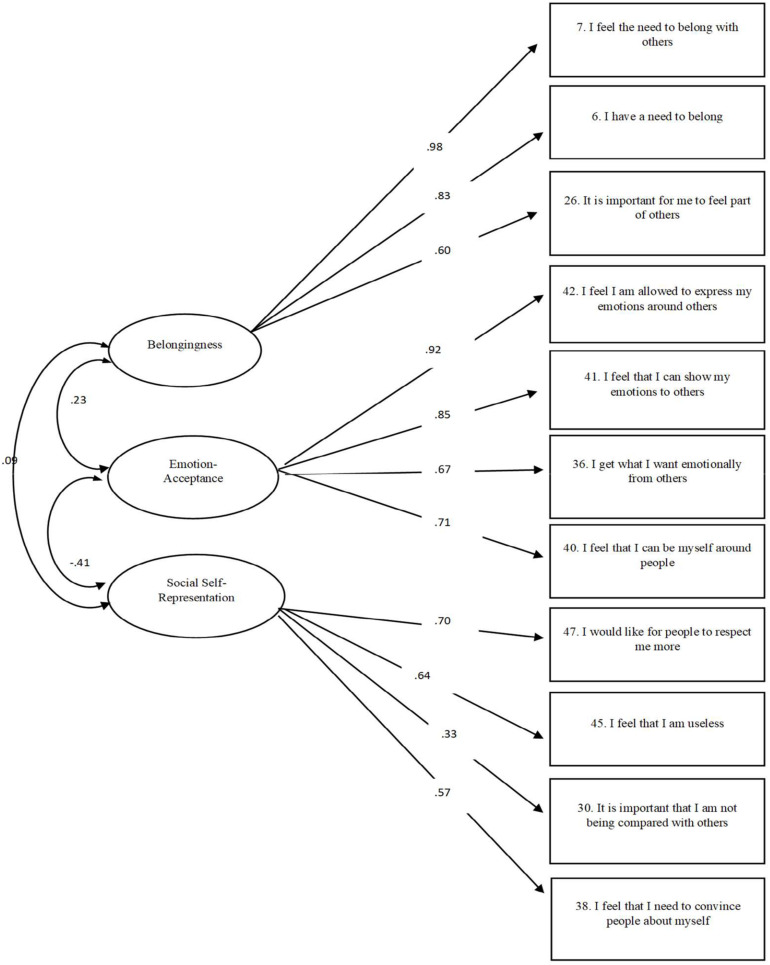
Three-factors model of Belongingness, emotion-acceptance, and Social Self-Representation, Emotional-Acceptance, and Social Self-Representation.

We performed a *CFA* with *AMOS* 26 from IBM to examine the three newly generated factors. Again, the missing data within the questionnaire was handled using “Estimate means and intercepts” allowing for ML estimation. No sample simulation was used. All three factors were allowed to correlate and correlations between error terms were not allowed to correlate. As before, we used the same fit indices and criterion to determine the goodness-of-fit of the model. As shown in Figure 1, the three-factors model confirmed that each item loading measures within limits and with significance of.30. Even though the χ*^2^* was significant χ*^2^* (41) = 143.78, *p* = 0.000, the χ*^2^/df* of 3.51 was within the range of acceptable (e.g., Jöreskog and Sörbom, 1993; Wan, 2002; Kline, 2005). Moreover, the other fit-indices such as the *CFI* = 0.937, the *IFI* = 0.938, and the *RMSEA* = 0.082, all indicated an acceptable to good fit of the model (e.g., Bentler and Bonett, 1980; Bentler, 1990; Fabrigar et al., 1999; Schumacker and Lomax, 2010) with an AIC of 215.78. Factor loadings ranged from λ = 0.33 to 0.98, and *M* = 0.71 indicating that the factors were well defined by their respective items (Gausel et al., 2012, 2016), with low to moderate correlations between the three different factors (ranging from *r* = −0.41, *p* = 0.001, to *r* = 0.23, *p* < 0.001). By such, we concluded that the exploratory factorial solution was replicated and validated with a *CFA* (see Table 2 for means, standard deviations, and correlations).

**TABLE 2:** Correlation on three constructs, and means and standard deviations.

Variables	Belongingness	Emotion-Acceptance	Social Self-Representation
Belongingness	–		
Emotion-Acceptance	0.29*	–	
Social Self-Representation	0.11	−0.31*	–
*M*	5.08	4.86	3.71
SD	1.26	1.14	1.11

*p < 0.05 (2-tailed).

The next steps in the scale validation procedure (Gausel et al., 2012, 2016) was to test the three-factorial structure up against other possible alternatives. First, we tested it up against a model where we allowed all the “Belongingness” items and all the “Social Self-Representation” items to load onto one factor, while the items of “Emotion-Acceptance” loaded onto a second factor. This model fit the data much worse than the three-factorial model (Δ *AIC* = 228.363, Δχ*^2^* (2) = 232.36, *p* < 0.000). Second, we tested the three-factorial structure up against a model where all items of “Belongingness” and “Emotion-Acceptance” loaded onto one factor, and “Social Self-Representation” items loaded onto a second factor. Again, this model fit much worse than the three-factorial model (Δ *AIC* = 504.184, Δχ*^2^* (2) = 508.18, *p* < 0.000). Third, we tested the three-factorial structure up against a model where all items of “Emotion-Acceptance” and “Social Self-Representation” loaded onto one factor, while the “Belongingness” items loaded onto a second factor. Again, this model fit much worse than the three-factorial model (Δ *AIC* = 153.537, Δχ*^2^* (2) = 157.537, *p* < 0.000). Finally, we tested the three-factorial structure up against a model where all items loaded onto one global factor. Like before, this model fit much worse than the three-factorial model (Δ *AIC* = 6439.276, Δχ*^2^* (2) = 6201.28, *p* < 0.000).

### Correlational Analyses of the Three Constructs

Looking at the correlations, there was a significant positive relationship between ‘belongingness’ and ‘emotion-acceptance’, *r* = 0.29, *p* < 0.001, such that the more belongingness needed, the more emotion-acceptance was needed, and vice versa. There was a weak, but significant, positive relationship between “belongingness” and “social self-representation,” *r* = 0.11, *p* = 0.034., such that the greater the need for belongingness, the greater the need to socially self-represent, and vice versa. Finally, there was a significant, negative relationship between “emotion-acceptance” and “social self-representation,” *r* = −0.31, *p* < 0.001, such that the greater the need for emotion acceptance, the less of a need to socially self-represent.

## Discussion

The goal of the current study was to test whether we were able to identify five variations of belongingness and 3 variations of acceptance. Somewhat to our disappointment, we realized that the 8 variations proposed were simply not possible to confirm. Using the approach to “scale validation” as suggested by Gausel et al. (2012, 2016), our CFA demonstrated that the first hypothesis was falsified. This means that the five variations of belongingness as we were able to identify in our reading of the literature: “Identity-proximity” (Kohut, 1971, 1977; Kohut et al., 1984), “emotion-sharing” (Lee and Robbins, 1995), “supportive-proximity” (e.g., Hill, 1987; Lazarus, 1991), “similarities of self and others” (Tajfel and Turner, 1979; Turner, 1999), and “environmental-satisfaction” (Bronfenbrenner, 1979; Bronfenbrenner and Ceci, 1994) were unsupported. The failure to confirm our hypothesis also meant that the three variations of “acceptance” in which we identified as: “Usefulness” (Rogers and Koch, 1959), “satisfactory” (Hayes, 1994), and “attitude to change” (Rogers and Koch, 1959; Linehan, 1993) were also unsupported.

We were also unable to receive support for our second hypothesis, that there should be enough shared similarities among the constructs to demonstrate a global “belongingness” factor (incorporating the five variations of belonging), and a global “acceptance” factor (incorporating the three variations of acceptance). By such, the *CFA* demonstrated that our second hypothesis, like the first one, was falsified. By such, the argumentation that there are two different needs, the need for belongingness and the need for acceptance (e.g., Baumeister and Leary, 1995; Shah, 2003), was not supported in these analyses. Nevertheless, the supplementary *CFA* demonstrated that belongingness and acceptance could not be made to “fit into” a unidimensional construct as long as the global “social need” factor failed to be supported. This latter finding therefore offers support to Baumeister and Leary’s (1995) argument that belongingness and acceptance are not the “same thing,” even though they are both involved in our understanding of oneself in relation to our social relationships, and it opens up for the reasoning of Lee and Robbins (1995) that the need to belong and the need for acceptance are elusive.

As we failed to receive support for our hypotheses, we had to return to a more data-driven approach and reach out to our participants in order to re-think and explore their responses through an Exploratory Factor Analysis (*EFA*). With this approach, three meaningful factors emerged in which we termed: “belongingness,” “emotion-acceptance,” and “social self-representation.” In regard of “belongingness,” the three items that made up this factor clearly tapped into the internal need to belong and the importance to feel part of others. This first factor therefore supports Kohut’s (1971, 1977), Kohut et al.’s (1984) view that there is an internal motivation, a drive to be needing “the other” in order to mirror oneself and to feel connectedness and alikeness in being part with the “other.” This factor also supports Lee and Robbins (1995), and Baumeister and Leary’s (1995) argumentation that the need to belong is fulfilled through reciprocal connectedness in relation to one’s social relationship. A similar view of the “belongingness” factor emerging from the *EFA* can be found in Brewer (1991) and Fiske’s (2004) theories, where the realization on one’s self-concept in terms of belongingness can only be meaningfully interpreted with reference to one’s attachment with social groups. By such, there is good support in the literature for our interpretation of the first factor of “belongingness.”

The second factor, “emotion-acceptance,” with its four items of being allowed to be oneself and to express one’s emotions around others, communicated a sense of acceptance focusing on being allowed to experience and own one’s emotions and to express them to others. This kind of emotion-acceptance can especially be found in Hill’s (1987) argumentation that a core motivation for a human motive is structured on the aim for continuous emotional support, and in Lazarus’s (1991) view that when people face a stressor, people typically try to communicate their emotions to others in order to receive emotional support and “get what they need” to cope with the stressor. This second factor of “emotion-acceptance” also lend support to Lee and Robbins (1995) that a secure development evolves from infancy to maturity through the ability to reciprocally share emotions. This need to share emotions and be accepted for one’s emotions bears a strong resemblance to Rogers and Koch’ (1959), Rogers’ (1961) view of the therapeutic dynamic between the client and the therapist where the therapist needs to be fully acceptive of the client’s emotions in order to allow the client to gain enough courage and trust to openly express emotions regardless of the emotions being “proper or not.” Similarly, this second factor lends support to Hayes (1994) view of acceptance as emotionally experiencing events openly and without defense regardless of the experiences being positive or negative. It is this need for acceptance of being allowed to openly express and show one’s emotions that Rogers and Koch (1959); Rogers (1961), and Linehan (1994) theorize as the keyways for the client to feel useful and to experience worth. In conclusion, there is much support in the literature for our interpretation of the second factor of “emotion-acceptance.”

The third factor, “social self-representation,” provided four items reflecting a value-based need to represent oneself socially as a person of worth in order to gain respect and to avoid being compared with others. This need for social self-representation illustrates a more complex interconnection and participation situated in the perspective of social representation and interactions (Ellemers et al., 2008), where one strives to present oneself favorably to avoid negative reactions from others due to social-image concerns (Gausel and Leach, 2011). This hinge to socially self-represent as someone to be respected and be worthy helps us construct an inner reality of how we perceive, feel, and think of ourselves, a process which is dependent on whether we receive positive or negative social feedback on our attempts to socially self-represent (Hardin and Higgins, 1996). In line with this, Leary et al. (2013, p. 3) stated that individuals that tend to “seek a larger number of relationships, worry about how they are valued by others, and put a great deal of effort into sustaining interpersonal relationships.” In support of the interpretation of this third factor, Cooley (1902) and Kohut (1971) underline how people try to portray a view of oneself in the eyes of others as someone to be liked and valued.

As the *EFA* had presented us with three factors that we were able to interpret as meaningful, and that we could relate to the literature, we decided to follow the recommendations of van Prooijen and van der Kloot (2001) to test whether it was possible to replicate the factorial solution proposed by the *EFA* with a *CFA*. Indeed, we managed to confirm the three-factorial structure suggested by the exploratory analysis. This model fit the data in an acceptable to good way and proved to be superior to all other alternatives to disentangle another factorial solution. Moreover, it proved to be superior to a solution where we collapsed all items onto a “social-need” factor. Like before, this latter finding supported Baumeister and Leary’s (1995) argument that belongingness and acceptance cannot be collapsed into the ‘same thing’.

Looking at the correlations, our result demonstrated that “belongingness” had a positive relationship with both “emotion-acceptance” and with “social self-representation.” For its relationship with emotion-acceptance, this means that the more one feels the need to belong, the more one feels the need to be accepted for one’s emotions and to be allowed to share them with others. This goes well with Baumeister and Leary’s (1995) argumentation that these two needs operate in conjunction, and it supports Lee and Robbins (1995) view that the need to belong is fulfilled through emotion-sharing and reciprocal connectedness.

For the positive relationship “belongingness” has with “social self-representation,” it means that these two needs operate together as well, so that the way one presents oneself socially must be involved in fulfilling the need for belongingness. Undoubtfully, if we present ourselves in a way that we think is socially approved of, there should be greater chance to secure belonging, instead of social condemnation (Gausel, 2013). Perhaps therefore, some people go to great lengths to agree with and follow others, even in destructive and immoral behavior, in order to feel that they belong and avoid being rejected (e.g., Leary et al., 2006; Smart Richman and Leary, 2009).

An interesting finding is the negative relationship between “emotion-acceptance” and “social self-representation,” meaning that the greater the need to be accepted for one’s emotions, the less of a need to present oneself in a favorable view in order to receive respect, and vice versa. This finding seems to indicate that if one is focused on trying to socially present oneself one cannot be open about who one is and what one feels, instead, one needs to be alert of which emotions to communicate (e.g., Hayes, 1994). By such, it lends support to the differentiation between socially acceptable emotions and socially unacceptable emotions and consequences of expressing them (Semin and Manstead, 1982; Meyerson, 1990; Tamir et al., 2016). Akin to social status (Fiske, 2010), social self-representation much like self-esteem (Cooley, 1902; Harter, 1993) are mutually in correspond to the positive and negative experiences that the self encounters with the environment and with others (e.g., Rogers and Koch, 1959; Rosenberg, 1965, 1979). Ultimately, not being able to fully be oneself and accepted for it by holding back on showing who one truly is, can accordingly result in the feeling of rejection and a risk of losing emotional connection within a social setting (e.g., Lynch, 1976; Relph, 1976; Canter, 1977).

## Possible Limitations

In response to the first and second hypothesis that failed, there is a plausible chance that the items we especially developed to reflect these eight variations of belongingness and acceptance were not representative of the variations we had identified. After all, we did not approach the theoreticians and asked them for their guidance in designing items tapping into their variation of the construct in which we identified – an approach suggested by Hernández et al. (2020). By such, one might say that the failed eight factor model, and the failed two factor model were caused by us developing “improper” items. However, we are convinced that the developed items represent the three variations of belongingness, emotion-acceptance and social self-representation in a meaningful information manner. Another limitation is the use of the English language. It could be that our findings are related to some artifact of the English language. Nevertheless, as long as the need to belong and feel accepted are theorized to be universal (e.g., Baumeister and Leary, 1995) there is ground to assume that one should be able to find similar results in other languages as well. Therefore, further studies can be examined based on similar items, yet with different languages, and different samples (e.g., Hernández et al., 2020). Some might say that a final limitation is our use of EFA and CFA on the same sample. However, we (and others) respectfully disagree with this viewpoint as other does as well (e.g., van Prooijen and van der Kloot, 2001; Schmitt, 2011). In line with this, van Prooijen and van der Kloot (2001) see it as a critical test of a factorial structure because if the *“CFA cannot confirm results of EFA on the same data, one cannot expect that CFA will confirm results of EFA in a different sample or population”* (p. 780). The main reason for this is that a CFA specified on an EFA in the same dataset operates under different conditions where two of the clearest differences are that EFA allows cross-loadings while CFA does not, and CFA provides fit of data, while EFA does not (Schmitt, 2011; Kline, 2015). Naturally, if the EFA cannot be replicated within the *same* sample, then there is less reason to expect factor replication with *different* datasets. This means that if the design and participant-pool are not 100% identical methodological variance will likely cause a failure of replication that is not caused by the factorial structure, but caused by methods, design and different people in the participant pool from the one study to the other (Fabrigar et al., 1999). It is precisely due to this dilemma that some suggest randomly splitting the file in two halves and then do EFA on the one and CFA on the other (Kline, 2015). However, in our view there is little reason to expect the two halves to be significantly different from each other. On the contrary, we believe there to be very good reason to expect the two halves to be *identical* as the participant pool is the same, the method is the same and the design is the same. Due to this, there is little reason to justify a split of the file in order to perform the EFA in the one half, then CFA in the other half under the belief that this is any more different than doing the same thing on a complete dataset. In conclusion, we believe our approach to be a sound approach while acknowledging that there are different views on EFA/CFA and how to understand a sample (Schmitt, 2011; Kline, 2013, 2015; Smith et al., 2016).

## Conclusion

In the ‘Breakfast club’, the five strangers interacted with each other in detention. At first, they presented themselves to each other socially as someone they hoped the others would find acceptable. However, as detention went along, they received a togetherness, a belongingness where each of the five found themselves to have less of a need to socially self-represent in an idealized way. Rather, they found the courage to express their emotions, to show the others who they really were; as fellow human beings with a burning need for belongingness and to be accepted based on their real self, of who they really are, and not a socially presented self. It is this realization motivating Brian’s declaration of the group as the “The Breakfast Club.”

## Data Availability Statement

The raw data supporting the conclusions of this article will be made available by the authors, without undue reservation.

## Ethics Statement

The studies involving human participants were reviewed and approved by FEK (Fakultetets Etiske Komite). The patients/participants provided their written informed consent to participate in this study.

## Author Contributions

SP did the design and analysis and contributed to the interpretation of the data, theoretical framework and write-up, and approved submission. NG contributed to the design and analysis, the interpretation of the data, theoretical framework and write-up, and approved submission. MH contributed to the interpretation of the data, design, theoretical framework and write-up, and approved submission. All authors contributed to the article and approved the submitted version.

## Conflict of Interest

The authors declare that the research was conducted in the absence of any commercial or financial relationships that could be construed as a potential conflict of interest.
